# Editorial: Brown Adipose Tissue: From Heat Production in Rodents to Metabolic Health in Humans

**DOI:** 10.3389/fendo.2021.739065

**Published:** 2021-07-26

**Authors:** Maria Chondronikola, Alexander Bartelt, Antonio Vidal-Puig, Kirsi A. Virtanen

**Affiliations:** ^1^ Department of Nutrition, University of California Davis, Davis, CA, United States; ^2^ Department of Nutritional Sciences and Dietetics, Harokopio University of Athens, Athens, Greece; ^3^ Institute for Cardiovascular Prevention, Ludwig-Maximilians-University, Munich, Germany; ^4^ German Center for Cardiovascular Research, Partner Site Munich Heart Alliance, Munich, Germany; ^5^ Institute for Diabetes and Cancer, Helmholtz Center Munich, German Research Center for Environmental Health, Neuherberg, Germany; ^6^ Department of Molecular Metabolism, Harvard T.H. Chan School of Public Health, Boston, MA, United States; ^7^ University of Cambridge Metabolic Research Laboratories, Institute of Metabolic Science, Medical Research Council, Metabolic Diseases Unit, Cambridge, United Kingdom; ^8^ Wellcome Trust Sanger Institute, Hinxton, United Kingdom; ^9^ Turku PET Centre, Turku University Hospital, Turku, Finland; ^10^ Department of Public Health and Clinical Nutrition, University of Eastern Finland, Kuopio, Finland; ^11^ Department of Endocrinology and Clinical Nutrition, Kuopio University Hospital, Kuopio, Finland

**Keywords:** adipose tissue, brown, metabolism and obesity, thermogenesis, adipose tissue beigeing

## Introduction

The unequivocal demonstration of functional brown adipose tissue (BAT) in adult humans (1–3) has led to a surge of scientific interest in its role in metabolic health. More recently, the discovery that white adipose tissue (WAT) can adopt a phenotype similar to BAT (a process also known as browning or beigeing of WAT) (4) has further fueled the hope to exploit the powers of thermogenic adipocytes against the obesity-related metabolic disorders. One of the unique characteristics of the thermogenic adipocytes is the large number of mitochondria enriched in uncoupling protein 1 (UCP1), which hotwires oxidative phosphorylation resulting in thermogenesis (5). Upon activation (in response to neuroendocrine, metabolic or dietary factors), thermogenic adipocytes increase their metabolic rate, oxygen consumption and accelerate glucose, lipid, and branched-chain amino acid metabolism (6). BAT has also been implicated as a potential endocrine organ, affecting the metabolic activity of distant tissues to coordinate whole-body metabolism (7). Although research efforts to this date have improved the current understanding of the regulation and metabolic significance of BAT and the thermogenic adipocytes, many questions remain to be answered. The manuscripts included in this Research Topic: i) summarize the current knowledge on the origins and plasticity of the thermogenic adipocytes and their potential role in metabolic health, ii) provide mechanistic insights on pathways implicated in thermogenesis, and iii) discuss current research gaps and how those can be addressed in the future.

## Molecular Mechanisms for the Maintenance of the Thermogenic Adipocytes

In this Research Topic, Rabbie summarizes the current knowledge on the origins of thermogenic adipocytes (8). This review also provides a comprehensive overview of the transcriptional and epigenetic factors involved in their development and maintenance. The author emphasizes the need for future research to improve the current understanding of the mechanisms involved in the bi-directional transition between the thermogenic adipocytes and the classical white adipocytes.

## The Role of BAT in Human Metabolism

The role of BAT in metabolic health is currently a topic of major scientific interest and debate. Pan et al. summarize the differences between rodent and human BAT, the methods for the assessment of human BAT, and the pathways regulating BAT thermogenesis (Pan et al.). McNeill et al. review the current evidence on substrate utilization by human BAT. Advanced medical imaging techniques and microdialysis have been used to assess substrate utilization in BAT. BAT has high capacity for plasma glucose and free and dietary fatty acid uptake especially in response to cold, while BAT also utilizes intracellular triglycerides for thermogenesis. Additionally, intermediate metabolites (i.e., pyruvate and lactate) and glutamate may fuel BAT thermogenesis and/or play a role in the regulation of BAT. Limitations of the currently available methods hinder the efforts to further understand substrate utilization by BAT further.

## Mechanistic Insights in the Regulation of Adipose Tissue Thermogenesis

### Electrical Neurostimulation (EN) for the Activation of BAT

Although the role of the nervous system and adrenergic signaling in the regulation of BAT metabolism is well-established (5), the currently available β-adrenergic agonists lack tissue-specificity, limiting their clinical utility due to cardiovascular side effects (8). Li et al. reported that EN of BAT in rodents increased BAT thermogenesis and decreased its intracellular lipid content, whereas β3-adrenergic blockade prevented the stimulation of BAT thermogenesis, suggesting that EN acts *via* beta3-adrenergic signaling (Li et al.). EN is a novel tissue-specific approach for the activation of BAT without the cardiovascular side effects of the systemic β-adrenergic stimulation.

### The Role of Macrophages in BAT Thermogenesis

Although resident immune cells in BAT have been implicated in the regulation of BAT, their role remains debatable (9). Fischer et al. reported that thermoneutrality led to pronounced macrophage infiltration of BAT, while cold exposure had the opposite effect (Fischer et al.). Moreover, exposure to thermoneutrality or room temperature conditions before a cold challenge did not affect the cold-induced transcriptomic response in BAT suggesting that the presence of macrophages in BAT may not affect thermogenesis. Future investigations are needed to assess the role of macrophages in BAT function comprehensively.

### Calsyntenin3β Regulation in BAT

Recent results from studies in rodents support that the neurotrophic and thermogenic calsyntenin3β-S100b axis regulates innervation in thermogenic adipose tissue (10). Plucinska et al. characterized the enrichment patterns of calsyntenin3β in various adipose tissue depots (Plucińska et al.). In humans, calsyntenin3β gene expression was higher in the perirenal multilocular BAT depot than the subcutaneous WAT, while its expression was positively associated with UCP1 expression. In rodents, calsyntenin3β gene expression was preferentially enriched in BAT and the enrichment pattern was sensitive to various physiological challenges (i.e., cold exposure, rewarming, and obesity). Calsyntenin3β is a promising target for the regulation of adipose-to-neuro axis.

## *In Vitro* Models for the Study of Human BAT

To this date, studies in animal models have been the primary source of knowledge on the cellular identity, plasticity, metabolic regulation and significance of the thermogenic adipocytes. However, this classic experimental approach does not account for interspecies differences between humans and rodents. Considering the high cost and limitations of clinical research, it is essential to establish efficient preclinical methods for studying of the thermogenic adipocytes. Smartly designed *in vitro* experiments can be used in conjunction with *in vivo* studies to address the open questions in the thermogenic adipocyte biology. Samuelson and Vidal-Puig reviewed the current methods studying of human BAT *in vitro* including immortalizing primary human brown adipocytes, multipotent stem cells, and human pluripotent stem cells and the advantages and limitations of each method (Samuelson and Vidal-Puig). Optimization of the current *in vitro* models to more closely recapitulate the *in vivo* micro-environment of the thermogenic adipocytes (e.g., innervation, vascularization, extracellular matrix, presence of other cells, and tissue architecture) is of paramount importance to accelerate scientific progress in realizing the role of BAT in the context of the obesity-related metabolic complications.

## Conclusion

BAT and thermogenic adipocytes constitute emerging targets against obesity and its related metabolic diseases that affect the well-being and quality of life of millions of people worldwide. To this date, the research progress on better understanding the origins, metabolic regulation and significance of BAT and the thermogenic adipocytes has been substantial. Nevertheless, many questions remain open. The most critical rate-limiting bottleneck hindering the scientific progress in the field of human BAT is the lack of tools to efficiently establish the signaling pathways regulating adipose tissue thermogenesis and to understand its metabolic significance at the tissue-specific and whole-body level. Answering those questions is critical to establish the thermogenic adipose tissue as a therapeutic target against metabolic disease.

## Author Contributions

MC and KV wrote the manuscript. AVP and AB edited the manuscript. All authors contributed to the article and approved the submitted version.

## Funding

MC is supported by the USDA National Institute of Food and Agriculture, Hatch project number CA-D-NTR-2618-H. AB was supported by the Deutsche Forschungsgemeinschaft Sonderforschungsbereich 1123 (B10), a Deutsches Zentrum für Herz-Kreislauf-Forschung Junior Research Group Grant, and the European Research Council Starting Grant PROTEOFIT.

## Conflict of Interest

The authors declare that the research was conducted in the absence of any commercial or financial relationships that could be construed as a potential conflict of interest.

## Publisher’s Note

All claims expressed in this article are solely those of the authors and do not necessarily represent those of their affiliated organizations, or those of the publisher, the editors and the reviewers. Any product that may be evaluated in this article, or claim that may be made by its manufacturer, is not guaranteed or endorsed by the publisher.
